# New York Observer

**Published:** 1871-11

**Authors:** 


### New York Observer.

The year 1872 will be jubilee year to the *New York Observer*, which was established in the beginning of 1823. This paper is one of the most influential in the country; and has acquired its influence by a rigid adherence to, and a fearless advocacy of, sound principles in Church and State. It has both a Religious and a Secular Department, kept distinct; and although not political or partisan in its character, it freely expresses and ably defends its views on matters of public policy. It has been for almost half a century a light in the Church and a pillar in the State. It will celebrate its jubilee by presenting to each one of its subscribers a New Year-Book—an encyclopædia of the most valuable information in regard to all those matters in the Church and in civil life which every one desires to have constantly at hand. New subscribers will receive the paper free until January 1st.

